# Traumatic Acute Colonic Intramural Hematoma: A Rare Entity and Successful Expectant Approach

**DOI:** 10.7759/cureus.9694

**Published:** 2020-08-12

**Authors:** Devarajan Jebin Aaron, Sandeep Bhattarai, Oseen Shaikh, Sarath Chandra Sistla

**Affiliations:** 1 Surgery, Jawaharlal Institute of Postgraduate Medical Education and Research, Puducherry, IND

**Keywords:** acute colonic haematoma, expectant management, pneumoperitoneum

## Abstract

Acute intramural hematoma in colon is a rare presentation following trauma. There are reports in literature of acute colonic hematoma following trauma, warfarin intake and in patient with coagulation disorders. In traumatic acute colonic intramural hematoma, most of the reported cases were managed surgically. Very few cases were successfully managed conservatively. We present a case of 28-year-old male who presented to the surgical emergency after two days of road traffic accident. After relevant investigations, he was found to have intramural hematoma of ascending colon, which was managed conservatively.

## Introduction

Acute colonic intramural hematoma is a rare case and only a few cases had been reported in the past. The common causes of acute intramural hematoma are trauma, warfarin, and coagulation disorders [1]. Blunt abdominal injuries causing colonic trauma is only 0.5-5% [1-3]. Among them, colonic intramural hematoma is very rare. The reason for the rarity in colon is supposedly due to the protective role of taenia coli, which can prevent blood diffusing in the bowel wall if there is a rupture of intramural vessels due to blunt trauma [4]. In traumatic acute colonic intramural hematoma, most of the reported cases were managed surgically. Very few cases are managed conservatively. Diagnosis is mainly by imaging with contrast-enhanced computed tomography (CECT) scan. If there are no abdominal signs and systemic signs of hypovolemia or sepsis, such patients can be managed conservatively.

## Case presentation

**Figure 1:**
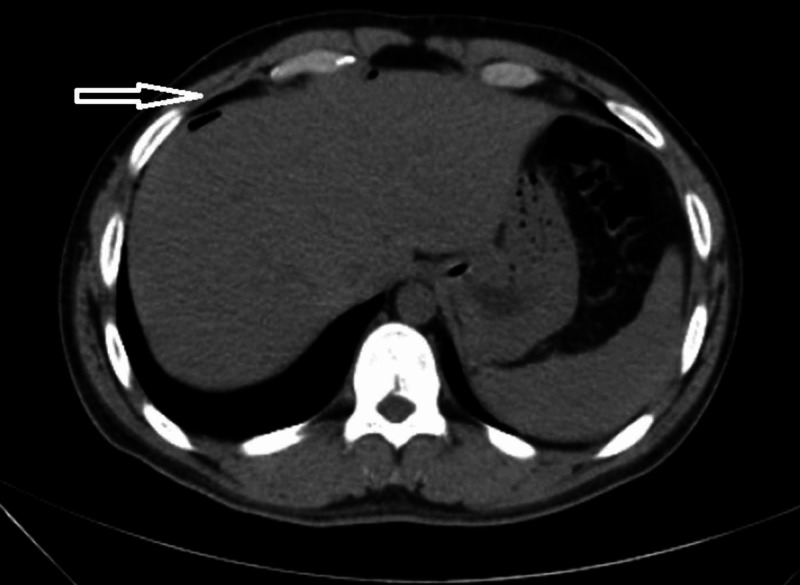
CT abdomen showing air pocket in subdiaphragmatic region suggestive of pneumoperitoneum (depicted by arrow).

**Figure 2:**
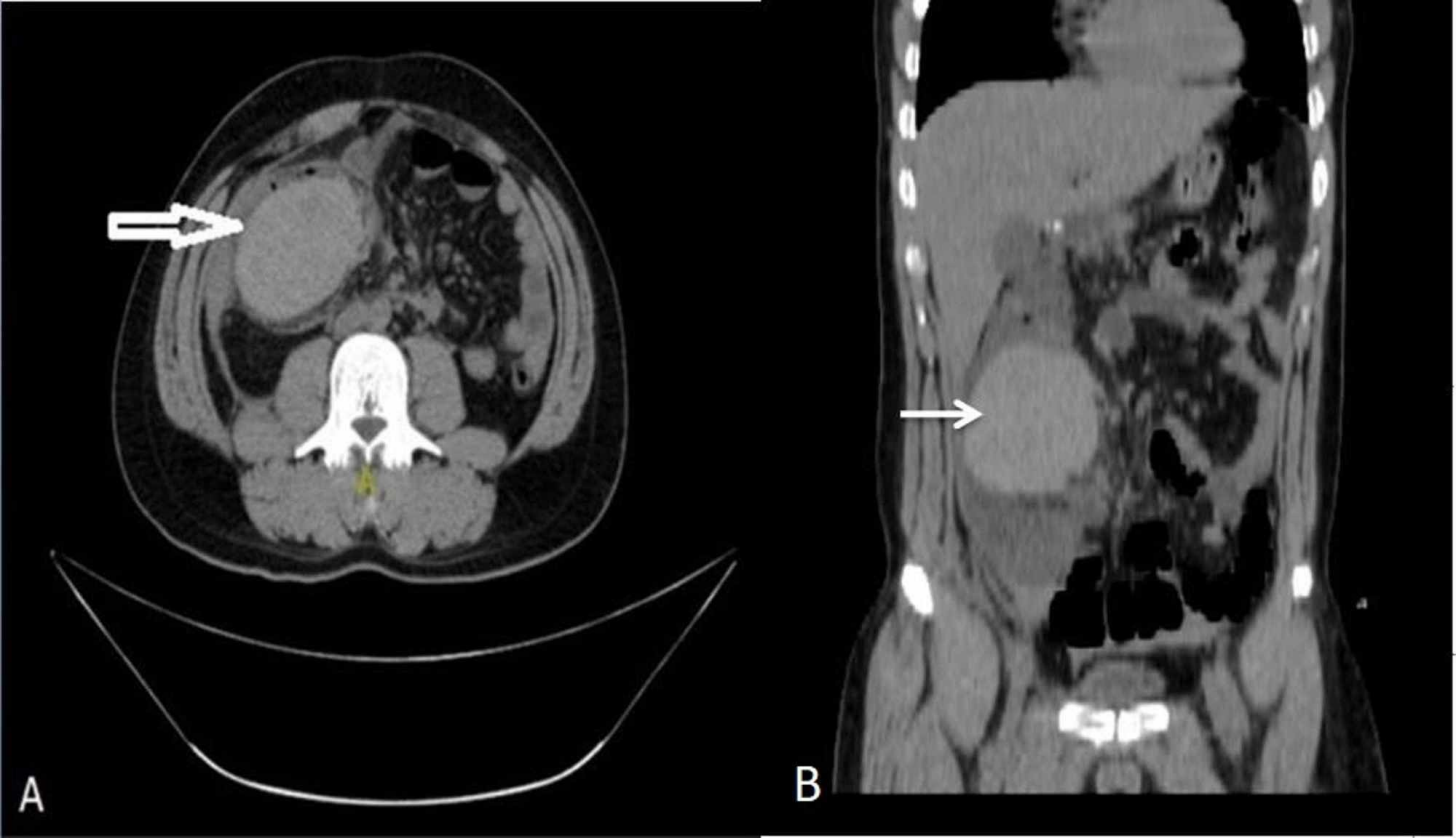
Contrast-enhanced computed tomography (CECT) abdomen showing intramural hematoma (arrow) of ascending colon. (A) Coronal view and (B) sagittal view.

**Figure 3:**
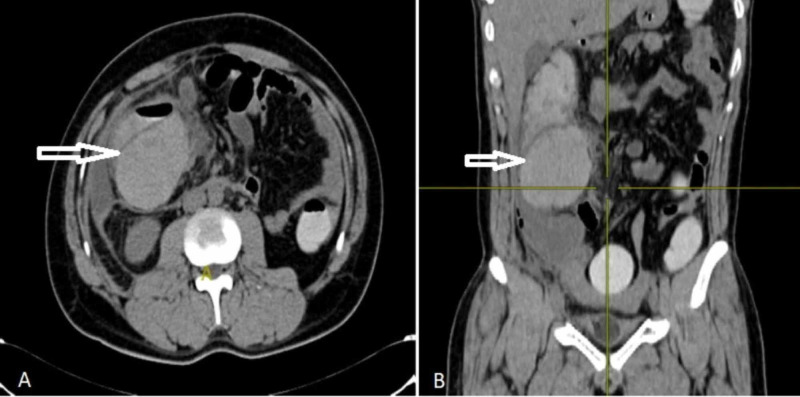
Contrast-enhanced computed tomography (CECT) abdomen with oral and rectal contrast showing intramural ascending colon hematoma. (A) Coronal view and (B) sagittal view.

A 28-year-old gentleman presented with an alleged history of a road traffic accident (RTA) two days before the presentation. He presented with injury over the right side of the abdomen and had continuous pricking type of pain in the right lower quadrant. He had vomiting and a few episodes of loose stools each day. He had no complaints of generalized abdominal distension, hematemesis, or melena. His vitals were stable. On abdominal examination, there was a tender palpable mass in the right iliac fossa. The digital rectal examination was normal. Chest X-ray erect didn’t show any free air under the diaphragm. Emergency ultrasonography (USG) showed minimal free fluid suggestive of hemoperitoneum. Contrast-enhanced computed tomography (CECT) of the abdomen was showing a small subdiaphragmatic air pocket suggestive of pneumoperitoneum (Figure 1). Also, there was large intramural hematoma in the ascending colon of size 9 x 6 x 9 cm, which was compressing the lumen of the ascending colon (Figure 2). There was a small serosal breach with the localized collection of small air pockets and minimal blood tracking retroperitoneally into the root of the sigmoid mesentery was noted. Since the patient had stable vitals with localized signs in the right lower quadrant, we had decided to manage him conservatively. After a day, oral and rectal contrast CECT abdomen was done and found to have no evidence contrast leak, no increase in the size of the hematoma, and no increase in pneumoperitoneum (Figure 3). The patient was continued on conservative management and was started on a normal diet. Serial USG was done intermittently and the hematoma was not found progressing. The patient was found to have a resolution of hematoma by USG of the abdomen over six weeks periods.

## Discussion

Acute colonic intramural hematoma rarely occurs following trauma to the abdomen. The first case of intestinal intramural hematoma was reported in 1855 by Thompson. Hughes et al. reported about 109 cases of post-traumatic acute intramural hematoma in 1977, in which only 4% of cases were colonic intramural hematoma [4]. Since 1950, there were 16 cases with traumatic colonic intramural hematoma, which was more common in ascending colon [5,6]. Cases were reported to be managed by both conservative and surgical methods. One particular case was managed endoscopically by using endoscopic clips and dual knife [7].

The pathophysiology for traumatic acute intramural hematoma was the shearing of the bowel wall layers due to decelerating forces or crushing forces, which causes rupture of terminal vessels entering into the bowel wall from mesentery, causing intramural hematoma [5]. The bowel regions, which were at the junction between retroperitoneally fixed and not fixed, were most commonly affected. The most commonly involved region of bowel with traumatic acute intramural hematoma was duodenum, followed by ascending colon/caecum [5,6].

Intramural hematoma can lead to acute and chronic complications. The acute complications are severe hemodynamic compromise due to blood loss by free rupture of hematoma into the peritoneum, gangrene of the involved bowel, intestinal obstruction, and intussusception [6]. The late complication is chronic stenotic lesion causing obstruction due to fibrosis. The colonic intramural hematoma was diagnosed using CECT abdomen. Barium studies show coiled spring or picket fence sign or stacked coin sign [5]. The patient can be followed up by using serial ultrasonography of the abdomen.

Conservative management can be considered if the patient is hemodynamically stable with no generalized peritoneal signs [8,9]. But, the patient should be monitored for danger signs, like hemoglobin drop, an increase in the size of the hematoma, and change in peritoneal signs. Surgical intervention to be done, if there is an active arterial bleed into hematoma seen as arterial blush in CECT arterial phase, and if generalized peritonitis signs are present. This patient reported in this study also had pneumoperitoneum, suggestive of hollow viscus perforation. He was managed conservatively as he had only localized peritoneal signs. Many case reports presented in the literature also favor conservative management for sealed bowel perforation.

## Conclusions

Traumatic acute colonic intramural hematoma is a rare presentation following blunt trauma abdomen, and treatment should be individualized according to the patient. Conservative management can be considered for patients who are hemodynamically stable with no generalized peritoneal signs. Contained colonic perforation can be managed conservatively in the absence of generalized peritonitis.
